# “INTERACTION BETWEEN BCL2 AND JUN B IN PRIMARY CUTANEOUS LYMPHOMA: A GENE ONTOLOGY APPROACH”: A COMMENT ON THE NEW APPROACH IN DERMATOLOGY

**DOI:** 10.4103/0019-5154.55648

**Published:** 2009

**Authors:** Viroj Wiwanitkit

**Affiliations:** *From the Dermatology Clinic, Wiwanitkit House, Bangkhae, Bangkok, Thailand 10160. E-mail: wviroj@yahoo.com*

Sir,

Generally, the new bioinformatics technique can be helpful in medical research. The hereby would like to discuss on an exampled application on cutaneous lymphoma, a rare clinical manifestation of systemic lymphoma that arises from the skin.[1] The author previously reported on the usage of gene ontology approach to clarify the interaction between Bcl2 and Jun B in primary cutaneous lymphoma via prediction for molecular function and biological process resulted from the combination between Bcl2 and Jun B.[2] According to this work,[2] with use of new tool, GoFigure,[3] the finding that Bcl2-Jun B is exactly similar to that of Jun B could be observed. Indeed, the application of gene ontology in other dermatological disorders can also be possible. The core concept of gene ontology is the searching for the recorded molecular function and biological process of each studied molecule. Then the combination of the two or more molecules due to molecular interaction is computational simulated and the difference and similarity pathways will be detected and used for constructed of finalized resulted ontology results. This technique is helpful in clarification of two or more molecules in the pathophysiological process of specific dermatological disorders. For example, the technique can be applied to assess the interaction of two molecules in pathogenesis of autoimmune dermatological disorder or cancerous dermatological disorder. However, due to the fact that this kind of study is an in silico study, further experimental studies are needed before making a conclusion on any topics.
